# Domain Shifts in Machine Learning Based Covid-19 Diagnosis From Blood Tests

**DOI:** 10.1007/s10916-022-01807-1

**Published:** 2022-03-29

**Authors:** Theresa Roland, Carl Böck, Thomas Tschoellitsch, Alexander Maletzky, Sepp Hochreiter, Jens Meier, Günter Klambauer

**Affiliations:** 1 ELLIS Unit Linz, LIT AI Lab, Institute for Machine Learning, Johannes Kepler University Linz, Linz, Austria; 2 Department of Anesthesiology and Critical Care Medicine, Kepler University Hospital GmbH, Johannes Kepler University Linz, Linz, Austria; 3 RISC Software GmbH, Hagenberg i.M., Austria

**Keywords:** Machine learning, Domain shift, COVID-19, Blood test

## Abstract

Many previous studies claim to have developed machine learning models that diagnose COVID-19 from blood tests. However, we hypothesize that changes in the underlying distribution of the data, so called domain shifts, affect the predictive performance and reliability and are a reason for the failure of such machine learning models in clinical application. Domain shifts can be caused, e.g., by changes in the disease prevalence (spreading or tested population), by refined RT-PCR testing procedures (way of taking samples, laboratory procedures), or by virus mutations. Therefore, machine learning models for diagnosing COVID-19 or other diseases may not be reliable and degrade in performance over time. We investigate whether domain shifts are present in COVID-19 datasets and how they affect machine learning methods. We further set out to estimate the mortality risk based on routinely acquired blood tests in a hospital setting throughout pandemics and under domain shifts. We reveal domain shifts by evaluating the models on a large-scale dataset with different assessment strategies, such as temporal validation. We present the novel finding that domain shifts strongly affect machine learning models for COVID-19 diagnosis and deteriorate their predictive performance and credibility. Therefore, frequent re-training and re-assessment are indispensable for robust models enabling clinical utility.

**Supplementary Information:**

The online version contains supplementary material available at 10.1007/s10916-022-01807-1.

## Introduction

Reverse transcription polymerase chain reaction (RT-PCR) [1] remains the gold standard test for the coronavirus disease 2019 (COVID-19) [2]. However, RT-PCR tests are expensive, time-consuming, and not suited for high-throughput or large-scale testing efforts. In contrast, antigen tests [3] are cheap and fast, but they come with considerably lower sensitivity than RT-PCR tests [4]. Instead of RT-PCR tests or antigen tests, routine blood tests can be automatically scanned for COVID-19: machine learning (ML) models can predict the diagnoses on the basis of blood tests, which are taken in the routine processes of the hospital. The routine blood tests are acquired anyway, therefore, no additional efforts are caused by screening with ML models. Routine screening of the blood tests would allow frequent, fast and broad testing at low cost, thus providing a powerful tool to reduce new outbreaks in the hospital [5, 6]. Especially in developing countries with limited testing capacities, the ML enhanced tests can evolve into an efficient tool in combating a pandemic.

ML methods offer very different ways to help confining the spread of infectious diseases [7–13], e.g., in developing vaccines and drugs for the treatment of COVID-19 [14–16]. COVID-19 diagnosis and the patient’s prognosis can be predicted from chest CT-scans, X-rays [17–25] or sound recordings of coughs or breathing [26–28]. Furthermore, it has been shown that ML models based on blood tests are capable of detecting COVID-19 infection [29–43]. Other outcomes, such as survival or admission to an intensive care unit can be predicted based on cheap and fast tests, such as blood tests [44–52].

In this study, we first reveal the *presence of domain shifts* in COVID-19-related blood test datasets. Second, we evaluate the ML models for prediction of COVID-19 diagnosis and mortality risk with different assessment strategies to demonstrate that these *domain shifts diminish the predictive performance*. Third, we compare the expected and actual performance to show how *model credibility is decreased by domain shifts*.

### Domain Shifts

Good generalization of ML models is only possible if the training data and future (test) data arise from the same underlying distribution. Deviations between training and test data distribution are a well known challenge in medical [53] and biological systems and in other real-world applications [54]. The failure of generalization on the test set and limited reliability of ML models in clinical settings has already been discussed in literature [55]. The negative effects and the necessity for countering these domain shifts in various complex biological systems have to be considered for ML models [56]. The necessity for critical appraisal and reporting of models for diagnosis and prognosis has been published in the context of the TRIPOD-AI guideline [57].

**Fig. 1:**
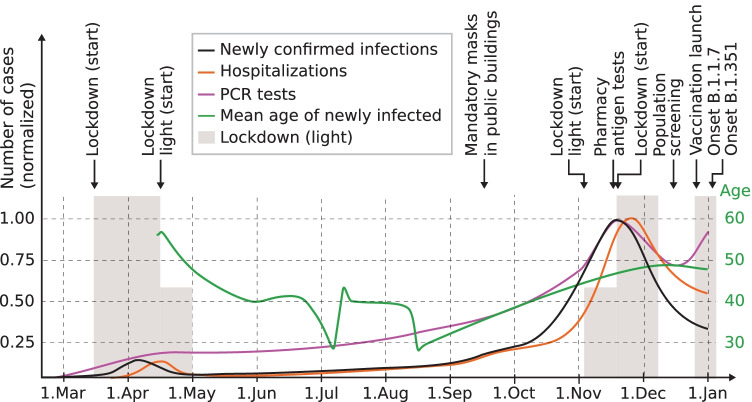
Examples of temporal domain shifts in COVID-19 datasets, which might diminish the ML model’s predictive performance over time. COVID-19 numbers in Austria over time, illustrating factors causing a temporal domain shift. The numbers are sketched according to data from the Austrian BMSGPK [58]

The same underlying distribution of training and future data also cannot be guaranteed during pandemics. Examples of potential domain shifts in COVID-19 related datasets are plotted in Fig. 1. Most of the previous COVID-19 ML studies evaluated their models by cross-validation, bootstrapping or fixed splits on randomly drawn samples [29–33, 37–43], which disregard changes in the underlying distribution over time, so-called domain shifts.

The domain shifts [54, 59, 60] can occur because of changes of the probability of observing a certain RT-PCR test result, which strongly changes during the pandemic. It can also change with the distribution of the blood test features, which are also affected by the overall pandemic course, but also, e.g., with the time of the year without connection to the pandemic [61]. The joint distribution of patient features and labels can change, e.g., with new virus mutations. Machine learning and statistical approaches model the probability to observe a certain RT-PCR test result given a patient. However, the RT-PCR test results might also be affected by changing test technologies or changing thresholds.

Neglecting and insufficiently countering these domain shifts can lead to undesired consequences and failures of the models. The domain shifts can lead to *degrading of predictive performance over time*, because standard ML approaches are unable to cope with domain shifts over time [54, 59, 60]. Further, the domain shifts can cause *unreliable performance estimates*. These performance estimates might be overoptimistic and can deviate significantly from the actual performance [62].

The ML models in our experiments do not require additional expensive features [32–34, 45–52]. The RT-PCR test results serve as the ground truth for the COVID-19 diagnosis (positive or negative) prediction. The in-hospital death is the label for the mortality (survivor or deceased) prediction of COVID-19 positive patients. The models are trained and evaluated on a large-scale dataset, which exceeds the dataset size of many small-scale studies [29–33, 43–46, 52] by far.

The findings of our work do not only apply to COVID-19 datasets, but also to future pandemics, other medical datasets and even to datasets from other fields, where domain shifts might play a role.

## Materials and Methods

Ethics approval for this study was obtained from the ethics committee of the Johannes Kepler University, Linz (approval number: 1104/2020). In our study, we analyze anonymized data only. The dataset was collected, pre-processed and the blood tests were merged with the RT-PCR tests.

As a first step, we plotted the statistics of the blood test parameters over time to visualize fluctuations of the statistics indicating the presence of domain shifts. To answer, whether domain shifts in the dataset cause degrading of predictive performance, we implemented different assessment strategies. To analyze the model credibility, a comparison of expected and actual performance was implemented and examined. Additional experiments and results are presented in the Supplementary Information.

### Dataset

**Fig. 2:**
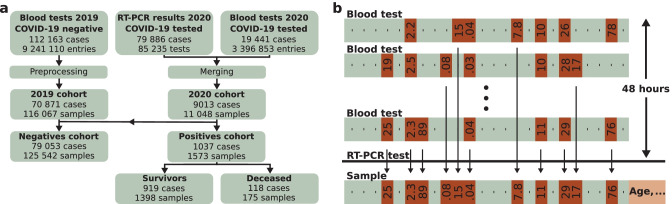
Large-scale COVID-19 dataset. **a**: A block diagram of the structure of the dataset. The blood tests from 2019 (blood tests 2019) are all negatives and are pre-processed to the *2019 cohort*. The COVID-19 RT-PCR test results and the blood tests are merged to the *2020 cohort*. The *negatives cohort* results from the *2019 cohort* (pre-pandemic samples) and the negative samples of the *2020 cohort*. The positive tested cases (*positives cohort*) are further divided to the cohort with the *survivors* and *deceased*. Note that one case can be in the *negatives* and *positives cohort* due to a change of the COVID-19 status. Multiple samples are obtained from one case, if RT-PCR and blood tests are measured repeatedly. **b**: Aggregation of the blood tests for the COVID-19 tested patients. The blood tests of the last 48 h before the COVID-19 test are merged to one sample. In case a feature is measured multiple times, the most recent one is inserted in the sample. Patient specific data, namely age, sex and hospital admission type, are added to the sample

The study is conducted on the dataset (Table 1) of the Kepler University Hospital, Med Campus III, Linz, Austria. The nature of the dataset corresponds neither perfectly to a cross-sectional study, since samples are taken at many different time-points, nor to a longitudinal study, since at each time-point a different set of samples is analyzed. Our analyses are based on blood tests, which are acquired in the routine process of the hospital. The features age, sex and hospital admission type (inpatient or outpatient) are added to the samples. If parameters in the blood tests are measured more than once, the most recent one is selected (Fig. 2). In case no COVID-19 test follows the blood test within 48 h in the *2020 cohort*, the blood test samples are discarded. Hence, the *2020* *cohort* is biased towards patients, who might already be suspect for being COVID-19 positive and therefore are tested. Additionally, all samples with a deviating RT-PCR test result within the next 48 h are discarded, as the label might be incorrect.

**Table 1:** Dataset with summary of patient characteristics

	N cases^a^	N positives	N negatives	Age (mean ± sd)	Sex (f/m), (f%)	Adm. type (i/o), (i%)^b^
Full dataset (*2019* and *2020 cohort*)	79 884	1037	79 053	53.4 ± 25.3	41 589/38 295 (52.1%)	50 727/29 157 (63.5%)
*2019 cohort* (pre-pandemic)	70 870	-	70 870	52.8 ± 25.1	36 934/33 936 (52.1%)	42 791/28 079 (60.4%)
*2020 cohort* (pandemic)	9014	1037	8183	58.0 ± 26.4	4655/4359 (51.6%)	7936/1078 (88.0%)
*Negatives cohort*	79 053	-	79 053	53.3 ± 25.4	41 213/37,840 (52.1%)	50 020/29,033 (63.3%)
*Positives cohort*	1037	1037	-	64.3 ± 20.2	455/582 (43.9%)	908/129 (87.6%)
*Survivors* (with COVID-19)	919	919	-	62.7 ± 20.5	417/502 (45.4%)	790/129 (86.0%)
*Deceased* (with COVID-19)	118	118	-	76.6 ± 11.8	38/80 (32.2%)	118/0 (100%)
March-October 2020 (training and validation cohort for prospective assessment)	6504	291	6277	57.0 ± 27.3	3416/3088 (52.5%)	5720/784 (87.9%)
November–December 2020 (test cohort for prospective assessment)	2636	785	1982	60.8 ± 24.1	1293/1343 (49.1%)	2335/301 (88.6%)

^a^Multiple samples can be obtained from one case. Therefore, one case can be contained in both, the *positives* and the *negatives cohort*, due to a change of the COVID-19 diagnoses, e.g., the patient might have been infected during the hospital stay, or the patient’s coronavirus load might have decreased, yielding a negative test result

^b^Adm. type: Admission type, i: inpatient, o: outpatient

Additionally, we incorporate pre-pandemic blood tests from the year 2019 as negatives to our dataset to cover a wide variety of COVID-19 negative blood tests (*2019 cohort*). The *2019 cohort* does not contain COVID-19 tests, therefore, blood tests with a temporal distance of less than 48 h are aggregated. A temporal distance of 48 h is selected such that the *2019 cohort* resembles the *2020 cohort*. The samples with less than 15 features are dropped from the dataset, all other available blood tests from the year 2019 are incorporated in the dataset. We assume that all patients in the year 2019 have been COVID-19 negative, because the virus has not been detected in Austria at this time. With a large, diverse dataset, the data distribution of the COVID-19 negative samples is broadly covered and learnt by the ML model. The distribution of the negative samples provided to the model during training has to be similar to the test data distribution for high predictive performance. During deployment, the model will be confronted with negative blood tests from a broad spectrum of different health scenarios, therefore, the *2019 cohort* is incorporated during training.

Before the selection of the 100 most frequent features, we include all available blood test parameters from the Med Campus III in Linz. This ranges from standard blood test parameters, such as leucocyte count up to blood tests for rare tropical diseases. Only the COVID-19 antibody tests are discarded from the dataset, as these might be directly related to the COVID-19 status. For the prediction of the COVID-19 diagnosis, the 100 most frequent features in the *2019 cohort* are selected as the feature set. For the mortality task these 100 most frequent features are selected based on the *positives cohort*. The number of measurements for each blood test parameter in the hospital is determined. The blood test parameters, which have been measured most frequently, are selected as input features for the ML models. Each sample requires a minimum of 15 features (minimum of any twelve blood test features and age, sex and hospital admission type). All other features and samples are discarded. Besides the measured blood test values, the selection of the acquired blood test parameters might also contain relevant information. Therefore, for each sample 100 additional binary entries are created, which indicate whether each of the features is missing or measured. The missing values are filled by median imputation. Hence, the models can be applied to blood tests with few measured values. In the full dataset (*2019 *and* 2020 cohort*) 58.0% and in the *positives cohort* 49.6% of the selected features are missing.

**Fig. 3:**
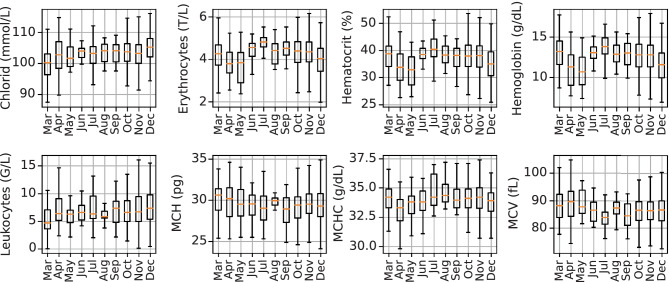
Statistics of blood test features of the *positives cohort*. The change of the statistics over time indicate a change of the underlying distribution and the presence of domain shifts. Abbreviations: mean cell hemoglobin (MCH), mean corpuscular hemoglobin concentration (MCHC), mean corpuscular volume (MCV)

Domain shifts are changes of the distribution over time, therefore, the mean, median and standard deviation, the first and third quantile of exemplary blood test features of the *positives cohort* are displayed in Fig. 3. Indeed, the statistics change over time, which indicate the presence of domain shifts. These eight features are the most frequently measured blood test features in the *positives cohort*.

### Machine Learning Methods and Model Selection

We investigate the capability of different ML model classes to predict the COVID-19 diagnoses and the mortality risk. To this end, the predictive performance of self-normalizing neural networks (SNN) [63], K-nearest neighbor (KNN), logistic regression (LR), support vector machine (SVM), random forest (RF) and extreme gradient boosting (XGB) are compared against each other. The pre-processing, training and evaluation is implemented in Python 3.8.3. In particular, the model classes RF, KNN and SVM are trained with the scikit-learn package 0.22.1. XGB is trained with the XGBClassifier from the Python package XGBoost 1.3.1. The SNN and LR are trained with Pytorch 1.5.0.

The hyperparameters are selected via grid-search on a validation set or via nested cross-validation to avoid a hyperparameter selection bias (Table S2). The training, validation and test splits are conducted on patient level, such that one patient only occurs in one of the sets and the dataset is Z-score normalized based on the mean and standard deviation of the training set.

The models are selected and evaluated based on the area under the receiver operating characteristic curve (ROC AUC) [64], which is a measure of the model’s discriminating power between the two classes and is in this case equivalent to the concordance-statistic (c-statistic) for binary outcomes [64]. Further, we report the area under the precision recall curve (PR AUC) [65] and we also calculate threshold-dependent metrics, where the classes are separated into positives and negatives, instead of probability estimates. These metrics are negative predictive value (NPV), positive predictive value (PPV), balanced accuracy (BACC), accuracy (ACC), sensitivity, specificity and the F1-score (F1) [66]. We additionally report the thresholds, which are determined on the validation set to achieve the intended NPV.

### Experiments for Model Performance under Domain Shift

In this section, we evaluate whether domain shifts diminish the predictive performance of ML models. A flow chart about the assessments is shown in the supplementary information (Fig. S1). Therefore, five modeling experiments with two prediction tasks and different assessment strategies are set up:

#### COVID-19 Diagnosis Prediction

i. assessed by random validation with pre-pandemic negatives.
  All patients are randomly shuffled and split regardless of the patient cohorts (60% training, 20% validation, 20% testing). Domain shifts are not considered in this experiment. This experiment is performed to obtain an estimate of the predictive performance if there were no domain shifts in the data. This also corresponds to the performance estimates provided in other studies [29–34, 37–43], which we hypothesize to be over-optimistic.
ii. assessed by random validation with recent negatives.
  The training and validation sets include the *2019 cohort* and 80% (60% training, 20% validation) of the *2020 cohort*. The test set comprises the remaining samples (20%) of the *2020 cohort*. Therefore, the performance is estimated on patients, who actually were tested for COVID-19. Domain shifts between the *2019 cohort* and the *2020 cohort* are considered. Domain shifts within the *2020 cohort* are not considered. This experiment is executed in order to reveal the effects of biases and domain shifts between the *2019* and *2020 cohort*.
iii. assessed by temporal validation.
  The training and validation sets include the *2019 cohort* and the *2020 cohort* before November (80% training, 20% validation). A prospective performance estimation is conducted for the test set with all samples from November and December 2020. By the temporal split, domain shifts over time are considered. In this experiment, it is investigated how the models would perform in real-world environment, where models can only be trained with data from the past and deployed on future data.

#### Mortality Prediction

iv. assessed by nested cross-validation.
  The training (60%), validation (20%) and test (20%) sets comprise the *positives cohort*, which are the positive cases from the *2020 cohort*. Due to the limited number of samples, predictive performance is estimated with five-fold nested cross validation. This experiment is conducted to show the performance estimates, when domain shifts over time within the *positives cohort* are not considered. We hypothesize, that these results, which correspond to the performance estimates in other studies [46–48], are over-optimistic.
v. assessed by temporal validation.
  The training and validation sets include the positive cases from 2020 before November (80% training, 20% validation). The test set comprises the cases from November and December. In this experiment, domain shifts over time are considered. In this experiment, by temporal validation, the performance of the models with consideration of the domain shifts is estimated.

The performance estimates obtained by these different assessment strategies are compared. If the underlying distribution of the data remains similar over time, the performance estimates by random cross-validation and temporal cross-validation must also be similar. If the performance estimates of (ii) are different from (i), then former and more recent negatives follow different distributions and the ML models are affected by the domain shifts. If performance estimates from (iii) are lower than those of (i) and (ii), the distribution of the data changes over time, hence indicating the presence and diminishing effects of domain shifts on predictive performance. Equally, changing performance estimates from (iv) to (v) indicate a domain shift over time. The binomial test [67] is used to check, whether the ML model’s (SNN, KNN, LR, SVM, RF, XGB) performance estimates in experiment (i) are equal to the estimates in experiment (ii). Similarly, we compare experiment (ii) with (iii) and (iv) with (v).

### Experiments for Model Credibility under Domain Shifts

In this experiment, we test whether domain shifts cause deviations of expected and actual performance. The predictive performance would remain similar without domain shifts, but in the presence of domain shifts, the performance could be significantly different and thus domain shifts may be exposed. If the expected and actual performance are different, the diminishing effect of domain shifts on model credibility are revealed.

**Fig. 4:**
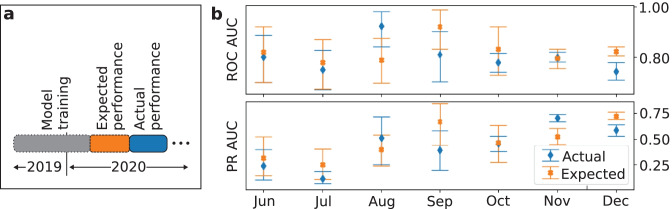
Comparison of expected and actual performance. **a**: The actual model performance is calculated for each month from June to December 2020 and the expected model performance is calculated on the respective previous month. The ROC AUCs of two subsequent months are compared, which correspond to expected and actual performance. **b**: The expected and actual performance with 95% confidence intervals. The expected and actual ROC AUC is significantly different in December and PR AUC differs significantly in November and December, showing the effect of the domain shifts on model credibility. Note that the PR AUC is sensitive to changes of prevalence

In this experiment, a standard ML approach is simulated in which a model is trained on data collected in a particular time-period (model training), then assessed on a hold-out set (expected performance) and then deployed (actual performance) (Fig. 4). For example, the deployment in December 2020 is simulated in the following way: First, an XGB model is trained (with the selected hyperparameters of experiment (iii)) on data from July 2019 until October 2020. The expected performance is then determined on data of November 2020. Then the actual performance of the model is evaluated on the subsequent month (December 2020). In other words, the ROC AUC metrics of two subsequent months are compared. The expected performance is determined with a temporal split, which might already be more credible than an expected performance assessed by random cross-validation. The 95% confidence intervals are determined via bootstrapping by sampling 1000 times with replacement.

## Results

### Model Performance under Domain Shifts

In general, ML models are capable of diagnosing COVID-19 and predicting mortality risk with high ROC AUC values. XGB and RF outperform other model classes in the COVID-19 diagnosis and in the mortality prediction. The comparison of evaluations on different cohorts expose domain shifts and their diminishing effect on predictive performance. Results are reported in terms of threshold-independent performance metrics for the comparison of the models (Tables 2 and 3) as well as threshold-dependent metrics (Tables S3, S4, S5, S6 and S7).

**Table 2:** Performance metrics of threshold-independent metrics for COVID-19 diagnosis prediction (experiment (i)-(iii)). The mean and the standard deviation ( ±) for the ROC AUC and PR AUC for the five random seeds are listed. Note that the PR AUC is dependent on the class prior, which changes with the different assessment strategies. E.g., the class prior in the test set in experiment (iii) is higher, because disease prevalence in the evaluation months November and December is higher. The performance estimates of a random estimator (RE) and the best feature (BF) are listed for comparison. The highest performance metrics per experiment are printed in bold

Model	**Experiment (i)**		**Experiment (ii)**		**Experiment (iii)**	
	ROC AUC	PR AUC	ROC AUC	PR AUC	ROC AUC	PR AUC
RE	0.5000 ± 0.0000	0.0124 ± 0.0000	0.5000 ± 0.0000	0.0822 ± 0.0000	0.5000 ± 0.0000	0.3162 ± 0.0000
BF	0.6745 ± 0.0000	0.0221 ± 0.0000	0.6774 ± 0.0000	0.3141 ± 0.0000	0.6623 ± 0.0000	0.5716 ± 0.0000
SNN	0.9567 ± 0.0025	0.4349 ± 0.0306	0.8998 ± 0.0044	0.5577 ± 0.0074	0.7836 ± 0.0053	0.6620 ± 0.0082
KNN	0.9071 ± 0.0000	0.3137 ± 0.0000	0.8432 ± 0.0000	0.4486 ± 0.0000	0.7209 ± 0.0000	0.5712 ± 0.0000
LR	0.9600 ± 0.0008	0.4126 ± 0.0145	0.8878 ± 0.0022	0.4770 ± 0.0086	0.7732 ± 0.0008	0.6467 ± 0.0059
SVM	0.9611 ± 0.0000	0.4268 ± 0.0000	0.9045 ± 0.0000	0.5573 ± 0.0000	0.7759 ± 0.0000	0.6387 ± 0.0000
RF	**0.9654** ± **0.0005**	0.5231 ± 0.0106	0.9138 ± 0.0025	0.5761 ± 0.0100	0.7957 ± 0.0025	0.6626 ± 0.0049
XGB	0.9629 ± 0.0000	**0.5558** ± **0.0000**	**0.9169** ± **0.0000**	**0.6216** ± **0.0000**	**0.8142** ± **0.0000**	**0.7077** ± **0.0000**

**Table 3:** Performance metrics of threshold-independent metrics for mortality prediction (experiment (iv)-(v)). The mean and the standard deviation ( ±) for the ROC AUC and PR AUC for the five random seeds are listed. Note that the PR AUC is dependent on the class prior, which changes with the different assessment strategies. The highest performance metrics per experiment are printed in bold

Model	**Experiment (iv)**		**Experiment (v)**	
	ROC AUC	PR AUC	ROC AUC	PR AUC
RE	0.5000 ± 0.0000	0.1592 ± 0.0351	0.5000 ± 0.0000	0.1320 ± 0.0000
BF	0.7599 ± 0.0748	0.4320 ± 0.1021	0.7483 ± 0.0000	0.3938 ± 0.0000
SNN	0.8656 ± 0.0356	0.5866 ± 0.1196	0.8478 ± 0.0053	0.4917 ± 0.0110
KNN	0.8207 ± 0.0550	0.5527 ± 0.1137	0.8272 ± 0.0000	0.4669 ± 0.0000
LR	0.8613 ± 0.0351	0.5555 ± 0.1281	0.8388 ± 0.0088	0.4784 ± 0.0173
SVM	0.8587 ± 0.0306	0.5679 ± 0.1010	0.8271 ± 0.0000	0.4185 ± 0.0001
RF	**0.8813** ± **0.0214**	**0.6267** ± **0.1065**	**0.8572** ± **0.0071**	**0.5556** ± **0.0127**
XGB	0.8501 ± 0.0210	0.5196 ± 0.1005	0.8038 ± 0.0000	0.4334 ± 0.0013

#### COVID-19 Diagnosis Prediction

i. assessed by random cross-validation with pre-pandemic negatives.
  In this experiment, the highest ROC AUC performance is achieved, however, domain shifts are not considered in the performance estimate. The threshold-dependent metrics for the RF for multiple thresholds are reported, which are determined by defining negative predictive values on the validation set (Table S3).
ii. assessed by random cross-validation with recent negatives.
  The test set of experiment (ii) only comprises cases from the year 2020, which have been tested for COVID-19 with an RT-PCR test. Pre-pandemic negatives are excluded from the test set and the model is evaluated on pandemic samples only, which causes a performance drop from experiment (i) to (ii) (P = 0.016), see Table 2.
iii. assessed by temporal cross-validation.
  In this experiment, the model is trained with samples until October and evaluated on samples from November and December. An additional performance drop in comparison to experiment (ii) (P = 0.016) is observed, which points to a domain shift over time which degrades predictive performance.

#### Mortality Prediction

iv. assessed by random cross-validation.
  The samples are randomly shuffled and a five-fold nested cross-validation is performed. Again, the threshold-dependent metrics are reported (Table S6).
v. assessed by temporal cross-validation.
  In this experiment, the model is trained with samples until October and evaluated on samples from November and December for mortality prediction of COVID-19 positive patients (*positives cohort*). The performance drops from experiment (iv) to (v) (P = 0.016), revealing a domain shift and over time for mortality prediction. The domain shifts over time again decrease the predictive performance.

The conducted experiments explore different levels of consideration of the domain shifts by different assessments. The evaluations are compared on the basis of ROC AUC as the PR AUC depends on the class prior, which varies in the different evaluation cohorts. The results expose the domain shifts and their diminishing effect on predictive performance, as the performance drops from experiment (i) to (ii) and even further to (iii), and also from experiment (iv) to (v). By comparing experiment (i) and (ii) we investigate if inclusion of pre-pandemic negatives in the test set leads to overoptimistic metrics, and indeed variations in the performance metrics can be observed. We attribute this to the fact that the *2020 cohort* comprises patients who are suspect for COVID-19, some might even have characteristic symptoms, which are reflected in the blood tests. We hypothesize, that patients with characteristic symptoms tend to have similar blood test parameters, independent of their actual COVID-19 status. Therefore, a classification of the samples in the *2020 cohort* is more difficult and potential biases between the *2019* and *2020 cohort* cannot be exploited. Domain shifts over time within the year 2020 are considered in experiment (iii), which leads to a further decrease in predictive performance. Same holds for the drop of the predictive performance due to prospective evaluation in the mortality prediction task from experiment (iv) to (v).

### Model Credibility under Domain Shifts

This experiment investigates the difference of the expected to the actual performance. The expected and actual results are compared for different simulated deployment times (June until December 2020) (Fig. 4). The expected performance is calculated on the respective preceding month (May until November). The expected ROC AUC is higher than the actual performance in most months (Fig. 4). The expected ROC AUC performance for December is significantly lower than the actual performance in December. The expected and actual PR AUC differ significantly in November and December. These results show the presence of a domain shift and thus there is a necessity for up-to-date assessments, otherwise the performance estimate is not trustworthy.

Credible and highly performant ML models for in-hospital applications require frequent re-training and re-assessments to combat the domain shift effects. Stronger weighting of more recent samples increases the predictive performance under domain shifts. More details on the methods and results to frequent re-training and stronger weighting of more recent samples are described in the Supplementary Information.

## Discussion

Our set of experiments exposes the presence of domain shifts in COVID-19 blood test datasets as well as their detrimental effect on ML models. These domain shifts were insufficiently considered in previous works, which might have led to poor performance or even failure of the ML models in clinical practice. Therefore, our results suggest that the model performance should be frequently re-assessed. An up-to-date temporal evaluation appears indispensable to avoid unexpected behavior. The model should be frequently re-trained and more recent samples should be weighted stronger to exploit newly acquired samples and, thus, to counter the domain shift effect (see supplementary information, section Weighting of Recent Samples). Frequent re-training from scratch is a simple and feasible solution to handle the domain shifts, as ML models, such as RF or XGB for tabular data can easily be trained with limited computational resources. A high re-training frequency leads to fast adaptation to domain shifts and further to accurate predictions and assessments, but it is also associated with high effort for the acquisition of new samples and re-training of the ML models. This trade-off has to be balanced when selecting the re-training frequency in the hospital. Further, methods to handle the domain shifts could be considered, such as stronger weighting of recent samples during training.

In this large-scale study, we trained and evaluated our models with more samples than most studies [29–33] and we exploited pre-pandemic negative samples, which vastly increases our dataset size. The ML models achieved high predictive performance, comparable to previous studies [30–32, 35, 47], although the results cannot be directly compared as our assessment procedure is more rigorous. Different assessment procedures within our study also yielded highly variable performance estimates. In accordance with previous studies [29, 30, 35, 42, 48], XGB or RF for COVID-19 diagnosis and RF for mortality prediction were found to perform best. For increased validity and comparability of published performance estimates of clinical prediction models, it is highly recommended that authors stick to guidelines, such as TRIPOD-AI, thereby increasing the quality of published works in the medical AI research community.

One limitation of our work could be that we did not evaluate the generalization of our model to other hospitals. A transfer of a COVID-19 diagnostic model should only be done with thorough re-assessments, as a domain shift between hospitals might be present. However, this is not part of our investigation.

By automatic scanning of all blood tests, a large number of patients can be tested for COVID-19, which would not be feasible with expensive and slow RT-PCR tests. The ML predictions could enhance the established testing strategies in the hospitals, thereby broadening the screening. For re-training, at least some recent blood tests with associated ground truth RT-PCR test results have to be acquired to allow countering the domain shifts.

Our findings about domain shifts are not only relevant for COVID-19 datasets, but also transfer to other medical tasks, or in general, other applications of ML, where domain shifts occur. By advancing this field of research, we want to increase patient safety and protect clinical staff and we wish to make a contribution in banning the pandemic.

## Supplementary Information

Below is the link to the electronic supplementary material.

Supplementary file1 (DOCX 865 kb)

## Data Availability

Data sharing requires ethics approval.

## Code Availability

Code to reproduce our study is provided on GitHub (https://github.com/ml-jku/covid).

## Abbreviations

ACC: Accuracy
Adm. type: Type of hospital admission
BACC: Balanced accuracy
BF: Best feature
BMSGPK: Federal Ministry of Social Affairs, Health, Care and Consumer Protection
COVID-19: Coronavirus disease
c-statistic: Concordance statistic
CT: Computer tomography
F1: F1-score
i: Inpatient
KNN: K-nearest neighbor
LR: Logistic regression
MCH: Mean cell hemoglobin
MCHC: Mean corpuscular hemoglobin concentration
MCV: Mean corpuscular volume
ML: Machine learning
NPV: Negative predictive value
o: Outpatient
PPV: Positive predictive value
PR AUC: Precision recall area under the curve
RE: Random estimator
RF: Random forest
ROC AUC: Receiver operating characteristic area under the curve
RT-PCR: Reverse-transcriptome polymerase chain reaction
SNN: Self normalizing neural network
SVM: Support vector machine
XGB: Extreme gradient boosting
