# Supplementary material for: Domain Shifts in Machine Learning Based Covid-19 Diagnosis From Blood Tests
Source: J Med Syst. 2022 Mar 29;46(5):23. doi: 10.1007/s10916-022-01807-1 (PMC8960704; doi:10.1007/s10916-022-01807-1)
Supplement: Supplementary file 1 — Supplementary file1 (DOCX 865 kb) [file 10916_2022_1807_MOESM1_ESM.docx]

# Supplementary Information

## Experiments for Model Performance under Domain Shifts

The experimental design of the different assessment strategies for COVID-19 diagnosis and mortality prediction are shown in Fig. S1.

| 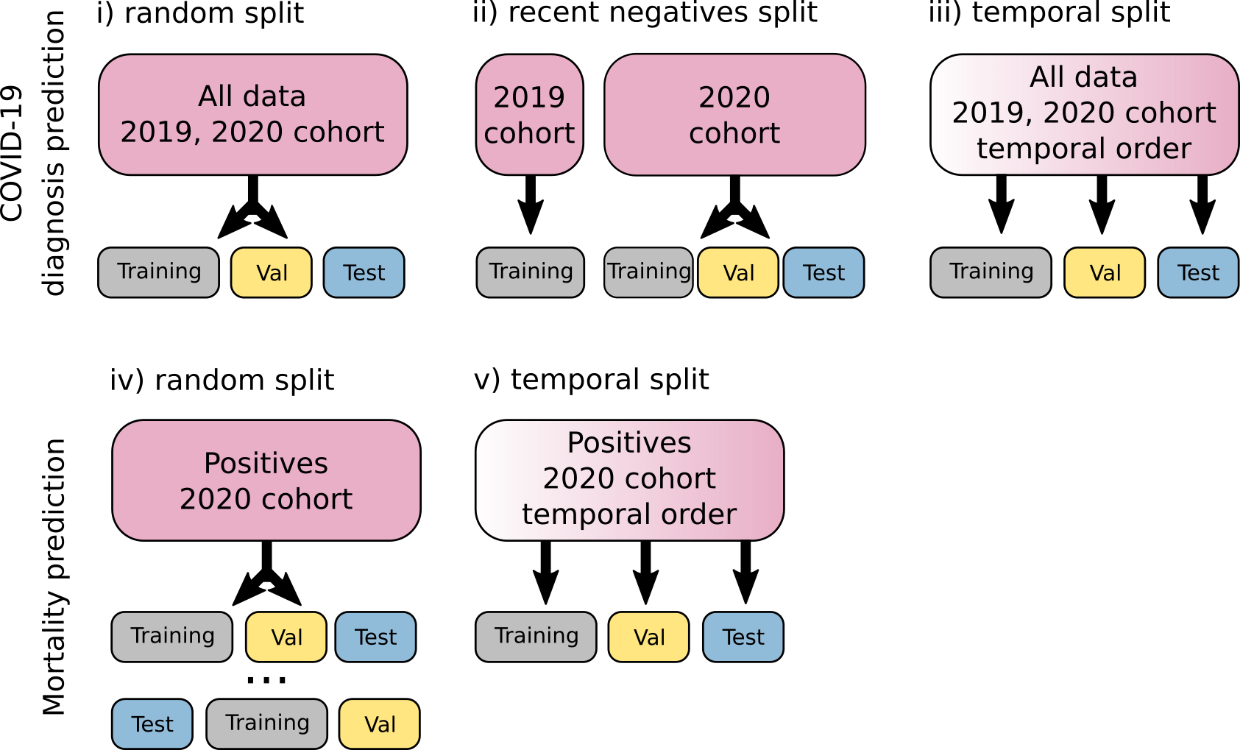  **Fig. S1** Flow chart of experimental design for different assessment strategies. The samples in experiment (i) and (iv) are randomly shuffled and then split into training, validation (val) and test set. The samples in experiment (ii) are separated into the *2019* and the *2020 cohort*. In experiment (iii) and (v) the samples are sorted in temporal order and split accordingly to consider the effects of domain shifts in the evaluation. |
| --- |

## Model Re-Training Frequency

### Methods

We investigate the effect of the model training frequency on the predictive performance. We evaluate the trained model on different numbers of subsequent months without re-training. We call this number of subsequent months the model training frequency, as it simulates the performance of not re-training the ML model for this period. Exemplary, we sketched a model training frequency of two months (Fig. S2). We concatenate the predictions as well as the ground truth label and calculate the ROC AUC and its 95% confidence interval calculated with bootstrapping 1000 times with replacement. We do not report PR AUC as it depends on the class prior, which changes over time with the disease prevalence.

| 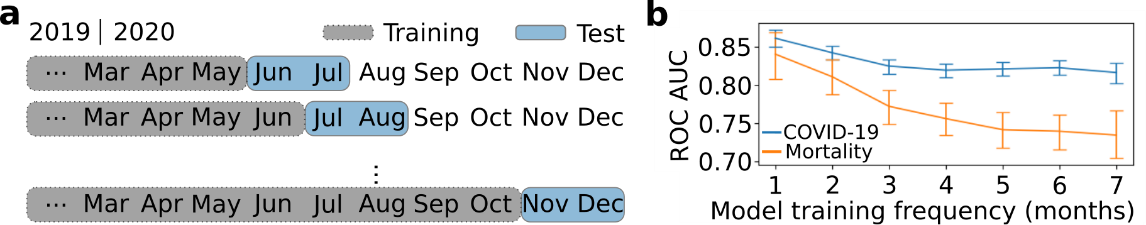  **Fig. S2** Model re-training frequency. **a**: Evaluation for a model training frequency of two months (exemplary). The model is evaluated on the two subsequent months after training. **b**: The effect of model training frequency on the ROC AUC. The mean and the 95% confidence intervals (error bars) of the ROC AUCs are plotted. The ROC AUC decreases with lower model training frequency. Hence, frequent re-training is indispensable for high predictive performance |
| --- |

### Results

The effect of the re-training frequency of the model on the ROC AUC is plotted (Fig. S2). The performance of the ML models increases with higher re-training frequency, thereby reducing the domain shift of the training to the test samples.

## Weighting of Recent Samples

### Methods

We investigate the effect of higher weights for more recent samples during training (Fig. S3). To this end, we define May until October as our validation months to select the optimal weighting and we evaluate the selection on November and December. We train the models with all available data before the respective validation month with the best hyperparameters determined in experiment (iii). With a one-sided, paired DeLong test^[[1]](#footnote-1)^ (pROC package 1.17.0.1 in R), we test our hypothesis that the ROC AUC increases when more recent samples are weighted higher than older samples, in comparison to all samples equally weighted.

| 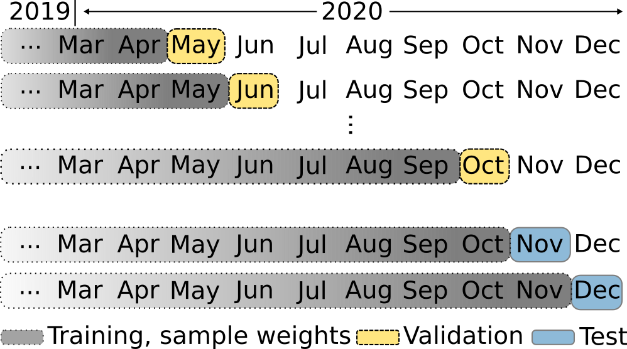  **Fig. S3** More recent samples are weighted higher in training to counter the domain shifts. The weighting is selected on the validation months starting from May until October. The selected weighting is evaluated on the test months November and December |
| --- |

We search for the optimal weights of the samples in dependence of their recency. We identify the best weighting by combining all listed options of weights of the *2019 cohort* and of the most recent, previous months on the validation set. We do a grid search over defined options for weighting the samples. We define weights for the *2019 cohort* and the weights for the *2020 cohort* depend on their recency. The default weight of the samples is 1. We restrict the *2019 cohort* weights to the set: {1, 0.1, 0.01, 0.001}, and the weights of the previous months to: {[1, 1, 1, 1], [1, 1, 1, 2], [1, 1, 2, 3], [1, 2, 3, 4], [2, 3, 4, 5]}, with the last entry in each square bracket being the weight of the last month, the second last of the second last month, and so forth. The best weighting parameters are selected on the validation set and tested on November and December.

### Results

To counter the performance drop caused by domain shifts, we propose to weight recent samples stronger during training of the COVID-19 diagnosis model (Fig. S3). On the validation set (May - October), we determine the best weighting in dependence of the sample recency. The highest performance gain on the validation set is achieved by setting the weight of the *2019 cohort* samples to 0.01 and the weight of the samples of the most recent month to 3, and the second last month to 2 ([1, 1, 2, 3]). Compared to weighting all samples equally, this significantly increases the ROC AUC on the validation set from 0.8118 (95% CI: 0.7849-0.8386) to 0.8502 (95% CI: 0.8271-0.8734) (P=9e-6). The selected weighting is tested on November and December, leading to a significant increase of the ROC AUC from 0.7996 (95% CI: 0.7831- 0.8162) to 0.8120 (95% CI: 0.796-0.828) (P=.005). Hence, including the *2019 cohort* samples with a low weight is better than weighting them equally to the *2020 cohort*. Further, a low weight on the *2019 cohort* is better than excluding the *2019 cohort* entirely from the training set. Additionally, it is beneficial to stronger weight recent samples from the *2020 cohort* during training.

## Features with Discriminating Capability

In Fig. S4, we present the most important features with discriminating capability, determined by calculating the ROC AUC of each individual feature as predictor (Table S1). We do not train a model for this purpose. The feature itself is the predictor for COVID-19 diagnosis or mortality risk.

| 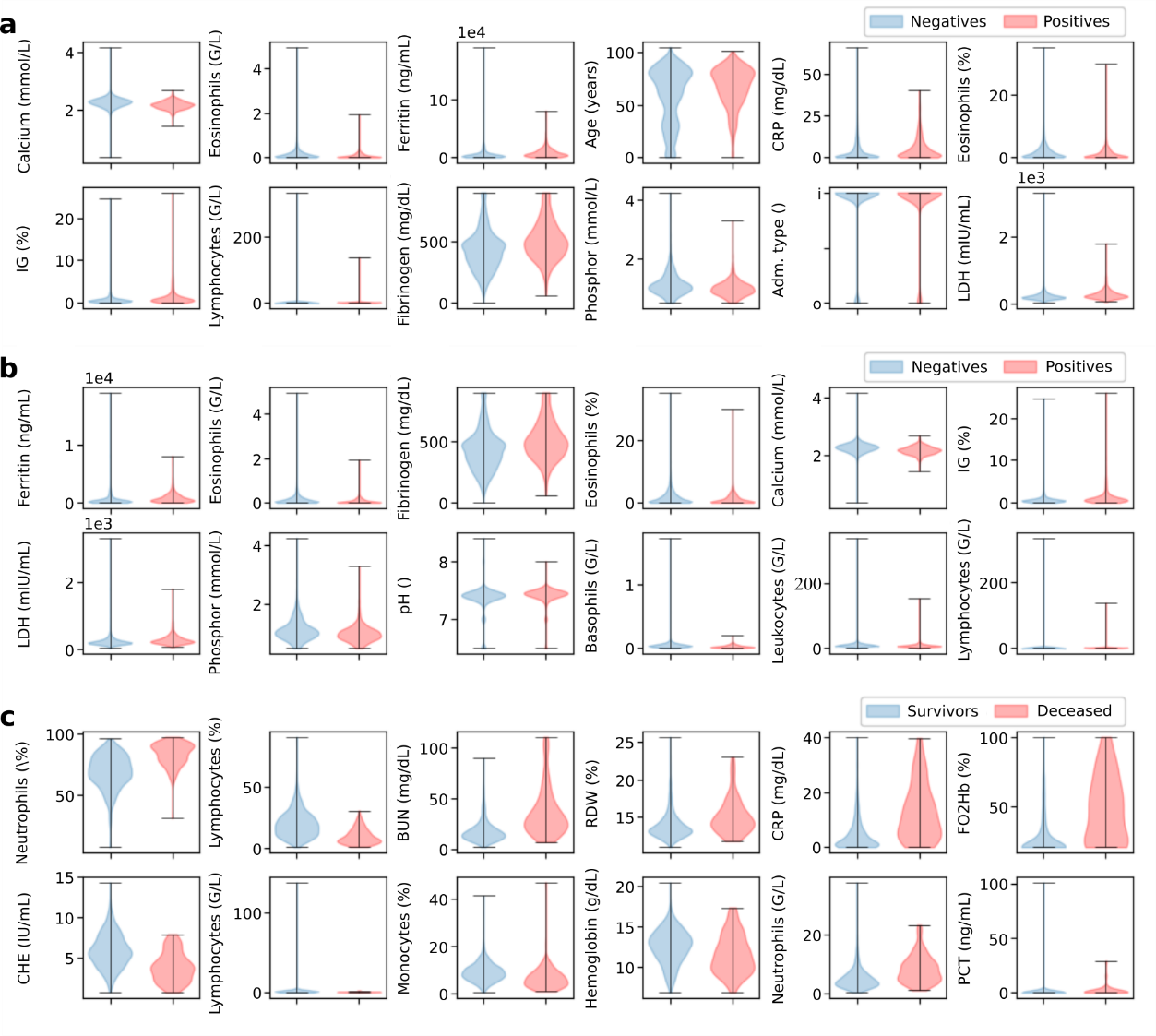  **Fig. S4** Features with discriminating capability in the **a**: *2019* and *2020 cohort* for COVID‑19 diagnosis prediction, **b**: *2020 cohort* for COVID-19 diagnosis prediction **c**: *positives* *cohort* (COVID-19 positive patients) for mortality prediction. Abbreviations: C-reactive protein (CRP), immature granulocytes (IG), type of hospital admission (Adm. type), inpatient (i), outpatient (o), lactate dehydrogenase (LDH), pH-value (pH), blood urea nitrogen (BUN), red cell distribution width (RDW), oxyhemoglobin fraction (FO2Hb), cholinesterase (CHE), procalcitonin (PCT) |
| --- |

| Table S1. Features with discriminating capability. The mean and standard deviation (±) for five different test sets at five random seeds are listed for experiment (iv). In all other experiments the test set is fixed.   \| Feature \| ROC AUC \| PR AUC \| \| --- \| --- \| --- \| \| **Experiment (i)** \| \| \| \| Calcium (−) \| 0.67 \| 0.02 \| \| AEC (−) \| 0.66 \| 0.11 \| \| Ferritin (+) \| 0.66 \| 0.08 \| \| Age (+) \| 0.66 \| 0.03 \| \| CRP (+) \| 0.66 \| 0.02 \| \| Eosinophils (−) \| 0.65 \| 0.12 \| \| IG (+) \| 0.65 \| 0.03 \| \| ALC (−) \| 0.65 \| 0.02 \| \| Fibrinogen (+) \| 0.65 \| 0.05 \| \| Phosphor (−) \| 0.64 \| 0.04 \| \| **Experiment (ii)** \| \| \| \| Ferritin (+) \| 0.68 \| 0.31 \| \| AEC (−) \| 0.66 \| 0.25 \| \| Fibrinogen (+) \| 0.66 \| 0.21 \| \| Eosinophils (−) \| 0.64 \| 0.25 \| \| Calcium (−) \| 0.64 \| 0.12 \| \| IG (+) \| 0.64 \| 0.16 \| \| LDH (+) \| 0.63 \| 0.13 \| \| Phosphor (−) \| 0.62 \| 0.17 \| \| pH (+) \| 0.61 \| 0.14 \| \| ABC (−) \| 0.60 \| 0.16 \| \| **Experiment (iii)** \| \| \| \| Ferritin (+) \| 0.66 \| 0.57 \| \| AEC (−) \| 0.64 \| 0.53 \| \| ABC (−) \| 0.63 \| 0.50 \| \| Fibrinogen (+) \| 0.62 \| 0.50 \| \| IG (+) \| 0.62 \| 0.45 \| \| Calcium (−) \| 0.62 \| 0.41 \| \| Eosinophils (−) \| 0.62 \| 0.51 \| \| Leukocytes (−) \| 0.61 \| 0.40 \| \| ALC (−) \| 0.61 \| 0.43 \| \| pH (+) \| 0.61 \| 0.45 \| \| **Experiment (iv)** \| \| \| \| Neutrophils (+) \| 0.76±0.03 \| 0.42±0.10 \| \| Lymphocytes (−) \| 0.76±0.04 \| 0.41±0.11 \| \| Blood Urea Nitrogen (+) \| 0.75±0.04 \| 0.38±0.12 \| \| RDW (+) \| 0.73±0.04 \| 0.35±0.10 \| \| CRP (+) \| 0.72±0.04 \| 0.39±0.09 \| \| Oxyhemoglobin Fraction (+) \| 0.70±0.05 \| 0.46±0.10 \| \| Cholinesterase (−) \| 0.70±0.03 \| 0.37±0.11 \| \| ALC (−) \| 0.69±0.05 \| 0.33±0.08 \| \| Monocytes (−) \| 0.68±0.04 \| 0.32±0.07 \| \| Hemoglobin (−) \| 0.68±0.02 \| 0.30±0.08 \| \| **Experiment (v)** \| \| \| \| Neutrophils (+) \| 0.75 \| 0.39 \| \| Lymphocytes (−) \| 0.74 \| 0.35 \| \| CRP (+) \| 0.71 \| 0.36 \| \| Oxyhemoglobin Fraction (+) \| 0.71 \| 0.42 \| \| Monocytes (−) \| 0.70 \| 0.35 \| \| Blood Urea Nitrogen (+) \| 0.70 \| 0.33 \| \| Neutrophils abs. (+) \| 0.69 \| 0.26 \| \| RDW (+) \| 0.68 \| 0.21 \| \| Procalcitonin (+) \| 0.68 \| 0.36 \| \| Cholinesterase (−) \| 0.68 \| 0.30 \| |
| --- | --- | --- | --- | --- | --- | --- | --- | --- | --- | --- | --- | --- | --- | --- | --- | --- | --- | --- | --- | --- | --- | --- | --- | --- | --- | --- | --- | --- | --- | --- | --- | --- | --- | --- | --- | --- | --- | --- | --- | --- | --- | --- | --- | --- | --- | --- | --- | --- | --- | --- | --- | --- | --- | --- | --- | --- | --- | --- | --- | --- | --- | --- | --- | --- | --- | --- | --- | --- | --- | --- | --- | --- | --- | --- | --- | --- | --- | --- | --- | --- | --- | --- | --- | --- | --- | --- | --- | --- | --- | --- | --- | --- | --- | --- | --- | --- | --- | --- | --- | --- | --- | --- | --- | --- | --- | --- | --- | --- | --- | --- | --- | --- | --- | --- | --- | --- | --- | --- | --- | --- | --- | --- | --- | --- | --- | --- | --- | --- | --- | --- | --- | --- | --- | --- | --- | --- | --- | --- | --- | --- | --- | --- | --- | --- | --- | --- | --- | --- | --- | --- | --- | --- | --- | --- | --- | --- | --- | --- | --- | --- | --- | --- | --- | --- | --- | --- | --- | --- |

| Table S2. Hyperparameters for grid search.   \| Model \| Hyperparameters \| \| --- \| --- \| \| SNN \| *lr*: {1e-3, 2e-4, 1e-4}, *n_val_stops*: {20}, *weight_decay*: {1e-5*}, intermediate_size:* {4, 16, 64}*, n_layers*: {1,3,6}*, alpha_dropout*: {0, 0.9}*, optimizer*: {Adam} \| \| KNN \| *n_neighbors*: {3,11,25,51,101,201,301}, *weights*: {uniform, distance} \| \| LR \| *lr*: {1e-2, 1e-3, 5e-4, 1e-4}, *n_val_stops*: {20}, *weight_decay*: {1e-5}, *optimizer*: {Adam} \| \| SVM (COVID-19) \| *class*: {LinearSVC}, *dual*: {False}, *class_weight*: {None, balanced} \| \| SVM (Mortality) \| *class*: {SVC}, *kernel*: {linear, poly, rbf, sigmoid, precomputed}, *probability*: {True}, *class_weight*: {None, balanced} \| \| RF \| *n_estimators*: {501}, *criterion*: {gini, entropy}, *max_depth*: {2, 8, 32, None}, *min_samples_split*: {2}, *min_samples_leaf*: {1,8,32}, *max_features*: {auto, log2, None}, *max_leaf_nodes*: {None}, *class_weight*: {balanced, None} \| \| XGB \| *objective*: {binary:logistic}, *booster*: {gbtree, gblinear, dart}, *eta*: {0.1, 0.3, 0.6}, *gamma*: {0}, *max_depth*: {2,6,32}, *scale_pos_weight*: {True, False}, *grow_polic*y: {depthwise, lossguide} \| |
| --- | --- | --- | --- | --- | --- | --- | --- | --- | --- | --- | --- | --- | --- | --- | --- | --- |
| Table S3. Performance metrics of threshold-dependent metrics of RF in experiment (i). Performance metrics on test set of RF for different thresholds selected on basis of the negative predictive value on the validation set (NPV val) of COVID-19 diagnosis prediction in experiment (i).   \| NPV val \| 0.999 \| 0.995 \| 0.990 \| 0.980 \| \| --- \| --- \| --- \| --- \| --- \| \| NPV \| 0.999*±*0.000 \| 0.995*±*0.000 \| 0.990*±*0.000 \| 0.988*±*0.000 \| \| PPV \| 0.066*±*0.002 \| 0.414*±*0.015 \| 0.823*±*0.014 \| 1.000*±*0.000 \| \| BACC \| 0.887*±*0.002 \| 0.812*±*0.005 \| 0.588*±*0.003 \| 0.501*±*0.001 \| \| ACC \| 0.834*±*0.007 \| 0.984*±*0.000 \| 0.989*±*0.000 \| 0.988*±*0.000 \| \| Sensitivity \| 0.941*±*0.004 \| 0.635*±*0.010 \| 0.176*±*0.006 \| 0.002*±*0.003 \| \| Specificity \| 0.832*±*0.007 \| 0.989*±*0.000 \| 1.000*±*0.000 \| 1.000*±*0.000 \| \| F1 \| 0.124*±*0.004 \| 0.501*±*0.009 \| 0.290*±*0.008 \| 0.004*±*0.006 \| \| Threshold \| 0.081*±*0.040 \| 0.444*±*0.098 \| 0.931*±*0.020 \| 0.995*±*0.001 \| |
|  |
| Table S4. Performance metrics of threshold-dependent metrics of RF in experiment (ii). Performance metrics on test set of RF for different thresholds selected on basis of the negative predictive value on the validation set (NPV val) of COVID-19 diagnosis prediction in experiment (ii).   \| NPV val \| 0.995 \| 0.990 \| 0.980 \| 0.975 \| 0.950 \| 0.900 \| \| --- \| --- \| --- \| --- \| --- \| --- \| --- \| \| NPV \| 0.998±0.000 \| 0.995±0.000 \| 0.981±0.000 \| 0.976±0.000 \| 0.955±0.000 \| 0. 918±0.000 \| \| PPV \| 0.117±0.005 \| 0.149±0.006 \| 0.289±0.010 \| 0.401±0.010 \| 0.687±0.022 \| 0.487±0.086 \| \| BACC \| 0.661±0.017 \| 0.737±0.011 \| 0.822±0.006 \| 0.826±0.004 \| 0.730±0.005 \| 0.502±0.001 \| \| ACC \| 0.382±0.033 \| 0.541±0.022 \| 0.818±0.008 \| 0.887±0.004 \| 0.939±0.002 \| 0.918±0.000 \| \| Sensitivity \| 0.994±0.005 \| 0.971±0.003 \| 0.826±0.007 \| 0.752±0.006 \| 0.479±0.009 \| 0.005±0.003 \| \| Specificity \| 0.328±0.037 \| 0.502±0.024 \| 0.818±0.008 \| 0.899±0.004 \| 0.980±0.002 \| 1.000±0.000 \| \| F1 \| 0.210±0.009 \| 0.258±0.009 \| 0.428±0.012 \| 0.523±0.009 \| 0.565±0.011 \| 0.011±0.005 \| \| Threshold \| 0.012±0.005 \| 0.027±0.011 \| 0.094±0.029 \| 0.151±0.047 \| 0.600±0.121 \| 0.979±0.019 \| |
| Table S5. Performance metrics of threshold-dependent metrics of RF in experiment (iii). Performance metrics on test set of RF for different thresholds selected on basis of the negative predictive value on the validation set (NPV val) of COVID-19 diagnosis prediction in experiment (iii). NPV val and NPV deviate because of domain shifts between the validation and test set.   \| NPV val \| 0.999 \| 0.995 \| \| --- \| --- \| --- \| \| NPV \| 0.836±0.003 \| 0.684±0.000 \| \| PPV \| 0.612±0.011 \| 0.938±0.108 \| \| BACC \| 0.500±0.002 \| 0.732±0.000 \| \| ACC \| 0.760±0.005 \| 0.684±0.000 \| \| Sensitivity \| 0.657±0.012 \| 0.001±0.001 \| \| Specificity \| 0.807±0.012 \| 1.000.±0.000 \| \| F1 \| 0.633±0.003 \| 0.002±0.002 \| \| Threshold \| 0.465±0.017 \| 0.985±0.004 \| |
| Table S6. Performance metrics of threshold-dependent metrics of RF in experiment (iv). Performance metrics on test set of RF for different thresholds selected on basis of the negative predictive value on the validation set (NPV val) of mortality prediction in experiment (iv).   \| NPV val \| 0.990 \| 0.980 \| 0.975 \| 0.950 \| 0.900 \| 0.850 \| \| --- \| --- \| --- \| --- \| --- \| --- \| --- \| \| NPV \| 0.973*±*0.021 \| 0.979*±*0.021 \| 0.971*±*0.022 \| 0.929*±*0.034 \| 0.867*±*0.041 \| 0.849*±*0.031 \| \| PPV \| 0.318*±*0.096 \| 0.299*±*0.109 \| 0.369*±*0.156 \| 0.523*±*0.161 \| 0.789*±*0.173 \| 1.000*±*0.000 \| \| BACC \| 0.748*±*0.023 \| 0.746*±*0.030 \| 0.775*±*0.032 \| 0.748*±*0.041 \| 0.596*±*0.063 \| 0.527*±*0.014 \| \| ACC \| 0.629*±*0.086 \| 0.609*±*0.101 \| 0.681*±*0.105 \| 0.822*±*0.085 \| 0.859*±*0.033 \| 0.850*±*0.030 \| \| Sensitivity \| 0.921*±*0.062 \| 0.937*±*0.064 \| 0.905*±* 0.077 \| 0.634*±*0.181 \| 0.206*±*0.141 \| 0.055*±*0.029 \| \| Specificity \| 0.575*±*0.107 \| 0.554*±*0.121 \| 0.644*±*0.136 \| 0.862*±*0.127 \| 0.985*±*0.016 \| 1.000*±*0.000 \| \| F1 \| 0.460*±*0.091 \| 0.439*±*0.107 \| 0.498*±*0.125 \| 0.536*±*0.097 \| 0.290*±*0.155 \| 0.103*±*0.053 \| \| Threshold \| 0.146*±*0.070 \| 0.151*±*0.062 \| 0.169*±*0.067 \| 0.332*±*0.106 \| 0.592*±*0.072 \| 0.793*±*0.089 \| |
| Table S7. Performance metrics of threshold-dependent metrics of RF in experiment (v). Performance metrics on test set of RF for different thresholds selected on basis of the negative predictive value on the validation set (NPV val) of COVID-19 diagnosis prediction in experiment (v). NPV val and NPV deviate because of domain shifts between the validation and test set.   \| NPV val \| 0.980 \| 0.950 \| 0.900 \| 0.850 \| 0.800 \| 0.500 \| \| --- \| --- \| --- \| --- \| --- \| --- \| --- \| \| NPV \| 0.977±0.006 \| 0.960±0.008 \| 0.946±0.002 \| 0.905±0.007 \| 0.870±0.003 \| 0. 868±0.000 \| \| PPV \| 0.235±0.017 \| 0.333±0.056 \| 0.407±0.023 \| 0.707±0.017 \| 0.944±0.079 \| 1.000±0.000 \| \| BACC \| 0.729±0.014 \| 0.766±0.014 \| 0.765±0.007 \| 0.651±0.026 \| 0.508±0.012 \| 0.501±0.001 \| \| ACC \| 0.593±0.045 \| 0.750±0.069 \| 0.826±0.013 \| 0.892±0.003 \| 0.870±0.003 \| 0.868±0.000 \| \| Sensitivity \| 0.915±0.031 \| 0.789±0.064 \| 0.683±0.016 \| 0.324±0.056 \| 0.017±0.025 \| 0.001±0.003 \| \| Specificity \| 0.544±0.006 \| 0.743±0.089 \| 0.848±0.016 \| 0.979±0.005 \| 1.000±0.001 \| 1.000±0.000 \| \| F1 \| 0.374±0.020 \| 0.463±0.047 \| 0.509±0.017 \| 0.440±0.049 \| 0.033±0.046 \| 0.003±0.005 \| \| Threshold \| 0.176±0.021 \| 0.257±0.059 \| 0.318±0.028 \| 0.486±0.036 \| 0.733±0.035 \| 0.792±0.026 \| |

1. E. R. DeLong, D. M. DeLong and D. L. Clarke-Pearson, „Comparing the areas under two or more correlated receiver operating characteristic curves: a nonparametric approach“, *Biometrics,* 44, p. 837-845, 1988. doi: 10.2307/2531595 [↑](#footnote-ref-1)
